# Stroboscopically induced visual hallucinations: historical, phenomenological, and neurobiological perspectives

**DOI:** 10.1093/nc/niaf020

**Published:** 2025-08-07

**Authors:** Trevor Hewitt, Ioanna Amaya, Romy Beauté, Anil K Seth, Timo T Schmidt, David J Schwartzman

**Affiliations:** Sussex Centre for Consciousness Science, Department of Engineering and Informatics, University of Sussex, Falmer, Brighton, BN1 9QT, England; Neurocomputation and Neuroimaging Unit, Department of Education and Psychology, Freie Universität Berlin, Habelschwerdter Allee 45, Berlin, 14195, Germany; Einstein Center for Neurosciences Berlin, Charité – Universitätsmedizin Berlin, Charitéplatz 1, Berlin 10117, Germany; Berlin School of Mind and Brain, Humboldt-Universität zu Berlin, Unter den Linden 6, Berlin 10115, Germany; Sussex Centre for Consciousness Science, Department of Engineering and Informatics, University of Sussex, Falmer, Brighton, BN1 9QT, England; Sussex Centre for Consciousness Science, Department of Engineering and Informatics, University of Sussex, Falmer, Brighton, BN1 9QT, England; Program for Brain and Consciousness, Canadian Institute for Advanced Research (CIFAR), MaRS Centre, West Tower, 661 University Ave., Suite 505, Toronto, ON M5G 1M1, Canada; Neurocomputation and Neuroimaging Unit, Department of Education and Psychology, Freie Universität Berlin, Habelschwerdter Allee 45, Berlin, 14195, Germany; Sussex Centre for Consciousness Science, Department of Engineering and Informatics, University of Sussex, Falmer, Brighton, BN1 9QT, England

**Keywords:** consciousness, contents of consciousness, perception, hallucinations, phenomenology

## Abstract

Exposure to rapid and bright stroboscopic light has long been reported to induce vivid visual hallucinations of colour and geometric formations. This phenomenon was first documented by Purkinje over 200 years ago. Since then, significant progress has been made in understanding the effects of stroboscopic light and the experiences it induces through multiple waves of interest from the scientific, therapeutic, and broader cultural communities. Despite these advances, fundamental questions remain unanswered, including comprehensive characterizations of its phenomenology, its precise physiological origins, under which conditions it may lead to altered states of consciousness phenomena, and potential clinical or therapeutic applications. This narrative review provides a historical summary of research into stroboscopic light stimulation (SLS) alongside its use in recreation and lay-therapeutic contexts. It also discusses the phenomenology of these experiences, current perspectives on the potential neural mechanisms of stroboscopically induced experiences, and provides an outlook for future research in this field.

## Introduction

Commonly reported stroboscopically induced visual hallucinations (SIVHs) mainly consist of vivid experiences of colour and geometric patterns. Less frequently reported are complex hallucinations containing semantic content, as well as experiences associated with altered states of consciousness (ASCs) phenomena such as out-of-body experiences, changes in time perception, and emotional responses (Pari, 2021; Smythies, 1959b; Ter Meulen et al., 2009; Wade et al., 2001). In addition to being fascinating in their own right, SIVHs are of interest as a model for studying visual hallucinations (VHs) under laboratory conditions, allowing for a systematic investigation of their phenomenology and the neural mechanisms that underlie them (ffytche, 2008; Pearson et al., 2016; Rogers et al., 2021). Recent theoretical and neurophenomenological work has begun to elucidate the neural mechanisms underlying SIVHs, potentially providing insights into properties of the visual system (Amaya et al., 2024; Bressloff et al., 2002; ffytche, 2008; Rule et al., 2011).

Since the 1960s, stroboscopic light stimulation (SLS) and the experiences it induces have occupied an obscure yet fascinating role in culture, spawning a wide variety of SLS devices and experiences, some of which have been developed as works of art (see ‘Cultural and recreational use’ subsection). In addition, some practitioners have sought to use SLS experiences as a recreational or therapeutic tool, drawing on the parallels in experience with psychedelic substances (see ‘Therapeutic and medical applications of SLS’ subsection). This article summarizes the history of SIVHs in science, culture, and medicine, providing a comprehensive review of their phenomenology, as well as the proposed neural and psychological mechanisms that may underlie these experiences.

### Stroboscopic light stimulation: techniques and terminology

Hallucination-inducing SLS typically involves bright, periodic light flashing between ~ 5 and 50 Hz, most commonly viewed through closed eyes. The eyelids serve as a medium to diffuse the light across the visual field, but this can also be achieved through other methods, such as goggles which diffuse light or bright strobing computer screens (Reeder, 2022; Walach and Käseberg, 1998). Precise brightness thresholds for the induction of SIVHs are difficult to establish due to a variety of factors which may influence their induction, including the level of ambient light, individual differences, and how tightly a participant closes their eyes during stimulation. However, brightness levels in the range of 1000–5000 lumens reaching the closed eyelids has been shown to reliably elicit SIVHs (Amaya et al., 2023; Bartossek et al., 2021; Montgomery et al., 2024; Schwartzman et al., 2019a; Smythies, 1959b; Walter, 1953), although SIVHs may occasionally be experienced outside of this range. For comparison, an average household LED bulb emits only around 800 lumens, while a typical smartphone flashlight emits around 50 lumens. Safety measures must be taken to mitigate the risks associated with exposure to SLS, such as for people with epilepsy or sensitivities to bright lighting (see Box 1).

In the literature, SLS has been referred to using a range of terms such as ‘flicker light’ (Bartossek et al., 2021) or ‘intermittent photic stimulation’ (Hammer and Arkins, 1964; Kasteleijn-Nolst Trenité et al., 2012). We propose that the clearest term is ‘stroboscopic light stimulation’ (Schwartzman et al., 2019a; Ter Meulen et al., 2009) for its specificity in referring to light that flashes at controlled intervals. SLS most effectively induces VHs when spread evenly across the visual field, which is commonly achieved by applying SLS on closed eyes (Smythies, 1959b; Ter Meulen et al., 2009), although other methods using open eyes stimulation with diffusion goggles have also been applied (Shenyan et al., 2024). Some authors use the term ‘Ganzfeld flicker’ or ‘Ganzflicker’ to refer to open eyes stimulation, such as when using strobing computer screens (Königsmark et al., 2021; Reeder, 2022). The German term ‘Ganz’ refers to a uniform coverage of the whole visual field, and should not be confused with ‘Ganzfeld’ stimulation, a perceptual homogenization technique (also termed perceptual deprivation), which can also induce hallucinations, though with a different phenomenology (Schmidt and Prein, 2019; Shenyan et al., 2024; Wackermann et al., 2008). Other studies using strobing stimuli on computer screens include checkerboards and annuli which may also give rise to SIVHs with distinct phenomenology (Billock and Tsou, 2007; Cilia, 2022; Pearson et al., 2016). SLS is also distinct from other methods that induce visual phenomena which involve flickering stimuli, such as pattern induced flicker colours (Von Campenhausen and Schramme, 1995) or frequency tagging (Norcia et al., 2015; Nozaradan et al., 2018; Regan, 1966), which do not aim to induce VHs and likely involve different neural mechanisms.

Few VHs are reported with SLS < 5 Hz or above flicker fusion (Becker and Elliott, 2006), the latter being the frequency at which stroboscopic light becomes subjectively indistinguishable from constant light which may be ≥ 50 Hz depending on the conditions and participant (Mankowska et al., 2021). The periodicity of SLS has been shown to be highly relevant for the induction of SIVHs, which diminishes if flashes are presented aperiodically (Amaya et al., 2023). Alterations to aspects of SLS such as frequency, colour, duty cycle (the percentage of each period in which the light is on), and brightness are known to affect the reported phenomenology of the experience (see ‘Phenomenology’ section). While these parameters are typically held constant in laboratory experiments, recreational applications of SLS often manipulate these parameters to create more dynamic and varied SLS experiences.

Visual experiences induced by stroboscopic light also have been referred to by various terms including ‘hallucinations’ (Billock and Tsou 2007; Rogers et al. 2021; Amaya et al. 2023), ‘phosphenes’ (Rule et al. 2011; Ermentrout and Billock 2022), ‘imagery’ (Sumich et al. 2018; Shenyan et al. 2024), or ‘pseudohallucinations’ (Reeder 2022). We suggest that the most suitable term is ‘hallucination’, defined as sensory experiences that do not correspond to external stimuli as would normally be the case for an individual’s baseline waking perception (ffytche 2008; Telles-Correia et al. 2015; Leptourgos et al. 2020; Rogers et al. 2021). This avoids conflating SIVHs with other distinct phenomena such as mental imagery (Farah 1989) or entoptic phenomena (Sevšek and Lumi 2022). ‘Pseudohallucinations’ is a term used in psychiatry to refer to hallucinations, usually pathological in nature, where the patient is aware of the non-veridical nature of their hallucinatory percept (El-Mallakh and Walker 2010). It is rarely used in contexts where non-pathological hallucinations are purposefully induced such as with psychedelics or SLS. We also avoid the term ‘phosphenes’: this term is usually used to refer to visual experiences in the absence of stimulation with light (Cervetto et al., 2009; de Graaf et al., 2018; Grüsser and Hagner, 1990), which is distinct from the VHs induced by SLS.

Box 1: Safety Considerations for Human Exposure to Stroboscopic Light

The primary risk factor for serious adverse effects from SLS is photosensitive epilepsy, although not all individuals with epilepsy are susceptible. While photophobia is common in epilepsy, photosensitive epilepsy represents only a small subset of cases (Quirk et al., 1995). Its prevalence in the general population is low, with an annual incidence of approximately one case per 100,000 people (De Bittencourt, 2004; Quirk et al., 1995). Photosensitive epilepsy may be up to six times more prevalent in children and adolescents (aged 7–19 years) than in adults (Quirk et al., 1995), leading to an estimated lifetime prevalence of about one in 10,000 (De Bittencourt, 2004). Although epilepsy—including its photosensitive form—is heterogeneous, there is broad agreement that it has a heritable component, as it frequently occurs in multiple members of the same family (Perucca et al., 2020). Additionally, individuals with high anxiety find certain types of bright light uncomfortable, with stroboscopic lighting in particular being anxiety-inducing (Khorshid et al., 2021). Studies have also associated autism spectrum disorder with hypersensitivity to bright light: e.g. photophobia increases with autistic traits (Corbett et al., 2009). Furthermore, commonly reported external triggers for migraines include bright, intense light, flickering light with strongly contrasting light and shade (Schulte et al., 2015). However, it should be noted that while certain environmental stimuli are frequently reported as triggers for migraine pain, there is ongoing debate regarding how such stimuli influence the onset of migraines (Schulte et al., 2015).

### History of the use of SLS

**Figure 1:**
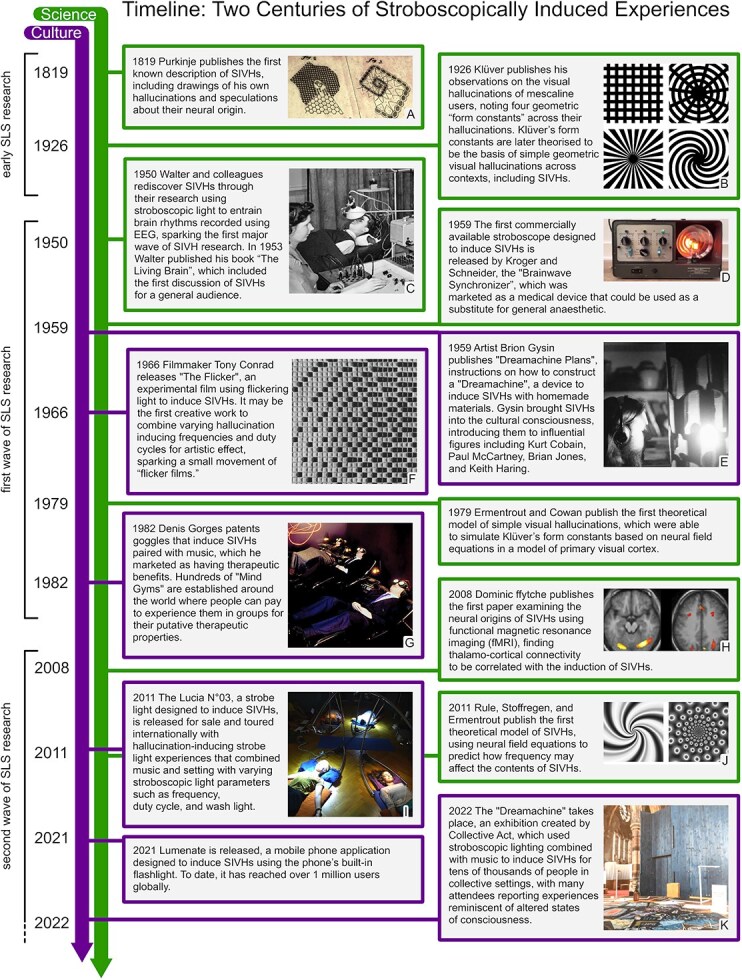
Timeline of the history of SIVHs. Key scientific and cultural milestones are shown. (A) Purkinje’s drawings of his SIVHs, the first known documentation of the phenomena (Purkinje, 1819) (B) A rendering of the four Klüver form constants (1926), which are geometric forms posited to make up simple geometric VHs, consisting of checkerboards (top left), targets (top right), tunnels (bottom left), and spirals (bottom right). (C) An early EEG experiment involving SLS at the laboratory of William Grey Walter. Such experiments eventually led to the rediscovery of SIVHs and the first wave of research into the phenomena (Başar, 2012). Photograph by Desmond Tripp. (D) Kroger and Schneider’s Brainwave Synchronizer, an early stroboscope sold as a medical device. Photograph by Paul Czerniak. (E) Gysin’s Dreamachine, a hallucination-inducing stroboscope that can be constructed from household materials which he invented, and which is often credited with introducing SIVHs to culture at large (Gatewood, 1970; Gysin, 1994). Photograph from Plutochaun, Wikimedia Commons. (F) A schematic of the arrangement of the dark and light frames used by Conrad for his short experimental film “The Flicker” (1966), created to induce SIVH experiences in the audience using a variety of SLS frequencies synchronised to sound (Hubby, 2016). Image courtesy the Tony Conrad Estate and Greene Naftali, New York. (G) One of Gorges’ ‘Mind Gyms’, where members of the general public could use SLS goggles for their posited therapeutic benefits (Ressmeyer, 1987). Photograph by Floris Leeuwenberg. (H) fMRI data of neural activations during SIVHs gathered by Dr Dominic ffytche, showing a correlation between thalamocortical activity and SIVHs (ffytche, 2008). Reprinted from Cortex Volume 44, Issue 8, Dominic ffytche, The hodology of hallucinations, Page 1075, Copyright 2008, with permission from Elsevier (I) An early group strobe session with the Lucia N°03 strobe light. Image courtesy of Light Attendance GmbH. (J) Rule, Stoffregen, and Ermentrout’s models of SIVHs, which simulate the content of SIVHs using neural field equations (2011). Images by Ethan Grove. (K) Collective Act’s Dreamachine, an experience which used stroboscopic lighting combined with music to induce SIVHs for tens of thousands of people within a collective setting, with many attendees reporting experiences reminiscent of ASCs (Collective Act, 2020). Image courtesy of Collective Act.

Research investigating the phenomenology and potential neuronal mechanisms of SIVHs dates back over 200 years (Fig. 1), beginning in the early 1800s with introspective reports of the phenomena (Brewster, 1834; Helmholtz, 1866; Purkinje, 1819). The ‘first wave’ of systematic investigation began in the 1950s, branching out from research into the electrophysiological (EEG) responses to stroboscopic light (Gebhard, 1943; Walter, 1953). Since the early 2000s, there has been a resurgence of interest in SLS, driven in part by the revival of psychedelic research (Tupper et al., 2015). This `second wave' has focused on gaining a better understanding of both the phenomenology and potential neural origins of SIVHs. Concurrently, SLS has also been utilized in cultural and therapeutic contexts since at least the 1950s.

To our knowledge, the first description of SIVHs was published by Purkinje in 1819 (see Box 2 for potential earlier instances). He described an introspective ‘experiment’ where he induced SIVHs by looking towards a candle flame and waving his hands in front of his closed eyes, and documented the geometric patterns he observed (Fig. 1A) (Purkinje, 1819). He discussed these observations alongside other ‘subjective phenomena’, such as mechanical and electrical phosphenes (in which visual experiences are induced by pressing on the eyes or electrically stimulating visual nerves), and speculated on the physiological origin of these phenomena. Soon after, the Scottish scientist David Brewster published his own exploration of SIVHs, including the first published observations on how SIVHs may vary with frequency (Brewster, 1834). For more than a century after Purkinje’s original publications, no influential studies on SIVH were conducted; there were only occasional mentions of SIVHs in relation to other phenomena involving flashing light, such as pattern-induced flicker colours (Brown, 1943; Gebhard, 1943; Helmholtz, 1866), until the advent of EEG reawakened interest in the topic.

### First-wave SLS research, 1900–2000

While early scientific discussions of various types of hallucinations primarily involved qualitative introspection (Brewster 1834; Helmholtz 1866), by the twentieth century, researchers began collecting qualitative reports of visual experiences from groups of participants and synthesizing the results to develop theories on the origin and perceptual contents of VHs (Klüver 1926; Smythies 1959b). In 1926, Heinrich Klüver published his influential manuscript ‘Mescal Visions and Eidetic Vision’ (Klüver 1926), in which he proposed a classification system for geometric VHs induced by mescaline, identifying four ‘form constants’ (see Fig. 1B). This classification system was later suggested to make up the majority of all reports of simple VHs across differing pathologies and induction methods (Ermentrout and Cowan 1979; Bressloff et al. 2002), including SIVHs (Rule et al. 2011).

In the mid-1900s, the advent of EEG sparked interest in stroboscopic light as a useful scientific tool for modulating EEG activity. Through this research, SIVHs were rediscovered by chance, which quickly led to a series of illuminating studies on the corresponding subjective effects of SLS. Most notably, William Grey Walter conducted pioneering studies after rediscovering the phenomena during his research (Walter 1953). In contrast to contemporary research into SIVHs, this period was marked by experiments with smaller sample sizes, more emphasis on qualitative research, and a wider variety of stroboscopic induction techniques (Gebhard 1943; Smythies 1959b; Remole 1971, 1973). Remole conducted a series of small experiments investigating how the luminance threshold for the perception of SIVHs changed with frequency (Remole 1974), colour of the light (Remole 1971), and monocular *versus* binocular SLS (Remole 1973). Smythies conducted a series of experiments aimed at describing and classifying the contents of SIVHs. He categorized the types of patterns and movement participants reported, noting the high inter-subject variability in subjective reports. He classified the geometric patterns into categories including unformed elements, rectilinear patterns, radial patterns, curvilinear patterns, and ‘formed images’ or complex hallucinations. Smythies’ thorough description and classification of the phenomena did not gain much traction in the scientific community. Instead, Klüver’s form constants became the dominant classification system used in modern studies of SIVHs (Rule et al. 2011; Mauro et al. 2015; Amaya et al. 2023) despite many reports of SIVHs containing geometric formations that extend beyond Klüver’s classification system (Smythies 1959b; Bressloff et al. 2002).

The history of SIVHs intermingles with much of the history of psychedelic research, and mirrors the rise and subsequent suppression of psychedelic research in the 1900s (Doblin et al. 2019), in that interest peaked in the 1960s but gradually waned over time. However, since the turn of the century, there has been a ‘second wave’ of interest in SIVHs, both culturally and within communities researching hallucinations, psychedelics, ASCs, and related phenomena.

### Second-wave SLS research, 2000 to present

The post-2000 second wave of strobe research was characterized by more controlled quantitative experiments (Pearson et al. 2016; Sumich et al. 2018; Shevelev et al. 2000), investigations into stroboscopically induced ASC phenomena (Schwartzman et al. 2019b; Bartossek et al. 2021; Montgomery et al. 2024), neurophenomenology (ffytche 2008; Amaya et al. 2024), and the use of SIVHs as a model to study simple VHs in other contexts (ffytche 2008; Pearson et al. 2016; Sumich et al. 2018; Reeder 2022). Large-scale quantitative phenomenological research has used stroboscopic frequency as an independent variable and has begun to elucidate more systematically how SIVHs may be affected by stroboscopic frequency (Shevelev et al. 2000; Herrmann and Elliott 2001; Becker and Elliott 2006; Allefeld et al. 2011; Shenyan et al. 2024).

Recent decades have also seen advances in the neurophenomenology of SIVHs, which aims to capture first-person descriptions of phenomena of interest (such as VHs) that are amenable to neurocognitive methodologies, including experiments linking phenomenological measures with brain imaging data from functional magnetic resonance imaging (fMRI) and EEG (Pütz et al. 2006; ffytche 2008; Wackermann et al. 2008; Becker et al. 2009; Mauro et al. 2015; Sumich et al. 2018; Schwartzman et al. 2019b; Heller et al. 2023; Amaya et al. 2024). MRI research has found thalamocortical activity to be implicated in the induction of SIVHs (ffytche 2008; Amaya et al. 2024) (see ‘Neural mechanisms’ section). Other studies have used EEG to investigate the electrophysiological correlates of SIVHs. A decrease in lower alpha band activity (8–10 Hz) has been observed to correlate with increased frequency of SIVHs reports (Becker et al. 2009; Sumich et al. 2018). Shevelev et al. (2000) found that different hallucinatory geometric forms were reported by participants when the SLS was synchronized to an individual’s peak intrinsic alpha frequency (IAF). Comparing SIVHs to psychedelic experiences, some experiments have examined whether the electrophysiological correlates of SLS-induced experiences resemble those associated with psychedelic experiences (Schwartzman et al. 2019a; Heller et al. 2023). While exposure to SLS has been found to correlate with increased neural signal diversity and other electrophysiological measures associated with psychedelic states, similar changes in EEG signals have also been observed at SLS frequencies which do not induce VHs (a common control condition in SIVH experiments). This suggests that the observed EEG effects may reflect the influence of the SLS itself, rather than being specific to the presence of VHs (Schwartzman et al. 2019a; Heller et al. 2023).

Another avenue of research has used SIVHs as a ‘model’ of VHs in various contexts (ffytche 2008; Pearson et al. 2016). SIVHs have been hypothesized to share broadly similar neural mechanisms with other types of simple VHs, and they can be much more easily induced and controlled in the laboratory with healthy participant populations compared to hallucinations arising from other aetiologies. Such experiments have used SIVHs as a tool to investigate the occurrence of VHs in Charles Bonnet syndrome (ffytche 2008), psychosis (Sumich et al. 2018), and Parkinson’s disease (Zarkali et al. 2019). Researchers have also used strobing stimuli on computer monitors to research SIVHs online and augmented with other visual stimuli (Billock and Tsou 2007; Pearson et al. 2016; Königsmark et al. 2021; Reeder 2022). Pearson and colleagues used a strobing ring on a computer screen to induce a simplified ‘model hallucination’, finding that the content of the SIVHs induced by this stimulus displayed properties of multistability, as well as replicating the frequency-dependent findings of other SIVH research (Pearson et al. 2016).

### Cultural and recreational use of SLS

While it is unknown when humans first began to experiment with SIVHs (see Box 2), the phenomenon entered into the cultural consciousness throughout the mid-1900s. This was largely due to the independent rediscoveries of the phenomena by William Grey Walter (see ‘First wave of SLS research’ section) and the beat generation artist Brion Gysin (Walter 1953; Ter Meulen et al. 2009; Hoptman 2010). While much was elucidated during the first wave of SLS research about its basic phenomenology and potential neural mechanisms, this work rarely left the confines of laboratories. In contrast, Gysin developed the ‘Dreamachine’, a stroboscope that could be constructed using household materials. This device would soon find its way into the homes of artists and bohemians throughout the 1970s and the 1980s, as well as in art exhibitions around the world (Gysin 1994; Geiger 2004; Hoptman 2010). Gysin’s was perhaps most significant for the influence he and his Dreamachine had on other artists, including musicians Kurt Cobain of Nirvana, Brian Jones of the Rolling Stones, and Paul McCartney of The Beatles, as well as other beat generation writers like Allen Ginsburg and William Burroughs (Gysin’s closest collaborator) and the artist Keith Haring (Geiger 2004; Sheehan 2008; Hoptman 2010; Sotheby’s 2020).

Influenced by both scientific and creative explorations of SIVHs during this period, structuralist filmmaker Tony Conrad experimented with using film projectors to induce SIVH experiences (Hubby 2016; Phelan 2015). His 1966 film ‘The Flicker’ may have been the first artistic work to vary the frequency and duty cycle of stroboscopic light over time in synchrony with music and sound in an attempt to produce a more structured and engaging hallucinatory experience (Conrad 1966; Fig. 1F). This notable innovation in the artistic use of SLS not only inspired a niche movement of experimental ‘flicker films’ (Gunning 2017) but also prefigures the contemporary cultural resurgence of interest in strobe which often uses sequences of synchronized SLS and sound.

Mirroring the two waves of interest in scientific research in SLS, artistic works surrounding SIVHs waned in the late 1900s before experiencing a resurgence of interest beginning in the 2000s. Gysin’s Dreamachine in particular has seen a renaissance of new art and commentary, inducing the publication of books (Gysin and Weiss 2001; Gysin and Férez Kuri 2003; Geiger 2004; Hoptman 2010), projects inspired by Gysin’s work and SIVHs (Hoptman 2010; October Gallery 2015; Collective Act 2020), a documentary film (Sheehan 2008), and recreations of Gysin’s Dreamachine available for purchase (Important Records 2024; Hafler Trio et al. 2016).

In the early 2000s, Proeckl and Winkler developed digitally controlled stroboscopes with adjustable frequency, duty cycle, and halogen wash lights, allowing for the creation and proliferation of stroboscopic sequences synchronized with music, marketed as the ‘Lucia N°03’ (Pearce et al., personal communication, 2024). This approach influenced the design of subsequent devices and platforms for curated SLS experiences (roXiva Ltd 2020; Pandora Star Ltd 2024). In 2021, the Lumenate mobile phone app was launched, utilizing the inbuilt flashlight LEDs on a smartphone as a strobe in combination with music and guided meditations, reaching over a million users globally (Lumenate Ltd 2021). In 2022, the UK-based art installation ‘Dreamachine’, inspired by Gysin’s original concept, used SLS and music in a curated setting to explore group experiences of SLS, introducing tens of thousands of attendees to SIVHs. Dreamachine was created and produced in collaboration with some of the present authors (T.H., A.K.S., and D.S.). Through the past two decades, hallucination-inducing stroboscopic light sequences have become a distinct artform, using the parameters of the strobe light itself alongside music, environment, and framing to create curated hallucinatory experiences.

Box 2: Rumours of Ancient SIVHs

The existence of SIVHs in ancient cultures is plausible but evidence is lacking. Gysin stated that Nostradamus would wave his hands in front of his eyes while staring at the sun to induce SIVHs, but no other sources have been found that support this story (Geiger 2004; Ter Meulen et al. 2009). Schumaker (1995) discussed the potential of fire-gazing as a source of ancient SIVHs, but these claims lack concrete anthropological evidence. Some sources have stated that the ancient Greek philosophers Ptoloemey and Apuleius observed SIVHs induced by light shining through the spokes of a spinning wheel (Michael Hutchison 1990); however, the original Greek texts appear to only discuss other optical phenomena involving spinning wheels, such as motion-induced colour mixing, with no clear reference to SIVHs (Harding and Jeavons 1994; Ptolemy and Smith 1996). It remains plausible that SIVHs may have existed in ancient cultures, given the importance of ASCs and VHs in many cultures (Hodgkinson 1996; Shepard 2005; Winkelman 2011) including the ancient Greeks (Ustinova 2009) and the ease with which SIVHs can be independently discovered. Several publications discuss apparent re-discoveries of SIVHs where the authors were unaware of the phenomena before encountering it (Brewster 1834; Gebhard 1943; Walter 1953; Gysin and Wilson 1985; Herrmann and Elliott 2001).

### Medical and therapeutic applications of SLS

Alongside decades of reports of ASC experiences among the cultural use of SLS, some researchers and practitioners have argued that the experiences induced by SLS may have medical or therapeutic benefits. Despite this long history of research and practice, these claims remain controversial. Besides SLS, other periodic stimulation methods—including repetitive transcranial magnetic stimulation and rhythmic sound—have been widely investigated by the medical community for their neurophysiological effects and therapeutic potential for disorders such as epilepsy, Parkinson’s disease, and depression (Dell’Osso et al. 2011; Specchio et al. 2011; Kim et al. 2016; Pereira et al. 2016; Wagle Shukla et al. 2016; Tian et al. 2021; Basu and Banerjee 2023; Huang et al. 2023). However, bright SLS is unique in the phenomenology it induces, which may, in turn, produce positive experiences that mediate therapeutic benefits (Roseman et al. 2018; Schwartzman et al. 2019a; Bartossek et al. 2021; Monroy and Keltner 2023). Indeed, within the field of psychedelic assisted therapy, it has been argued that the acute psychedelic experience itself may be pivotal for therapeutic outcomes, as opposed to such outcomes being purely pharmacological in origin (Roseman et al. 2018; Yaden and Griffiths 2021). It is unknown, however, if SLS-induced experiences share any mechanisms with psychedelic ASCs, or merely have suggestive phenomenological similarities. Research-backed medical benefits of SLS-induced experiences remain elusive.

One of the earliest medical applications of SLS-induced experiences occurred in 1959, when physician Dr William S. Kroger and inventor Sidney A. Schneider introduced the ‘Brainwave Synchronizer’. This device was marketed as a tool for inducing hypnotic states in order to reduce pain during surgery, positioning it as an alternative to anaesthesia (Bause 2010). However, the pain-reducing effects of the device were later attributed to participants’ expectations; pain relief only occurred when researchers informed participants that they should expect this effect, suggesting that the observed reduction in pain was not driven by SLS itself (Hammer and Arkins 1964). This finding, coupled with the device induced seizures in several patients, led to a sharp decrease in its popularity during the 1960s (Bause 2010). Since then, other researchers have continued to investigate the potential pain-reducing properties of SLS, although none have successfully isolated this effect from participants’ expectancies (Solomon 1985; Anderson 1989; Boersma and Gagnon 1992; Noton 2000; Horowitz and Telch 2007). Indeed, the potential influence of participant expectations—or, more generally, ‘demand characteristics’ (Orne 1962)—remains a challenge for SLS research today (see ‘Phenomenology of complex VHs and ASCs’ subsection).

In 1982, psychiatrist Denis Gorges patented the ‘Brain Wave Synchro-Energizer’, a set of goggles designed to induce SLS experiences, spawning a wave of practitioners who used such devices for their purported medical benefits, such as stress reduction and cognitive enhancement (Sutton 1988; Ravo 1992). Over the following decades, many similar devices were developed and sold, leading to the establishment of hundreds of ‘Mind Gyms’ worldwide, which provided SLS experiences to the general public. Researchers investigated the potential medical and wellbeing application of these devices for anxiety, phobias, depression, and memory among other conditions (Morse and Chow 1993; Berg 2006; Horowitz and Telch 2007; Berg and Siever 2009; Wolitzky-Taylor et al. 2008; Morse 2000; Ossebaard 2000; Wolitzky-Taylor and Telch 2010; Roberts et al. 2018). Results were mixed and often limited by flawed methodologies. The lack of robust evidence supporting the potential medical applications of these devices, combined with concerns over their potential to induce seizures, has led to controversy, including legal action against one manufacturer (Farley 1994). Although these devices never gained widespread medical acceptance, they continue to be used by some practitioners and members of the public for medical and wellbeing purposes to this day.

It remains unclear to what extent the phenomenological experiences induced by SLS may contribute to the treatment of a range of mental health conditions. In psychotherapeutic contexts, SLS has been used in a manner similar to, or in combination with, psychedelic-assisted therapy. Although this specific approach remains under-researched, proponents of the practice argue it can bring about therapeutic benefits in a manner similar to psychedelic-assisted therapy through the mechanism of acute ASC experiences. Recent research has reported promising response rates for psychedelic-assisted psychotherapy in controlled settings with screened participants (Luoma et al. 2020; Wheeler and Dyer 2020; Carhart-Harris et al. 2021; Ko et al. 2023), though not all such research has yielded positive results (Kupferschmidt 2024). Some studies further suggest that the acute psychedelic-induced visual experiences might mediate the long-term improvements in mental health, rather than being solely a result of pharmacological action (Roseman et al. 2018; Yaden and Griffiths 2021). Initial reports from psychotherapists have also found that SLS combined with psychedelics may induce more complex VHs, potentially intensifying the experience (Peretz et al. 1955; Grof 1996). Psychotherapists have also noted that the use of SLS in the context of psychedelic-assisted therapy has the further advantage of being easier to terminate than psychedelics, as SIVHs are rapidly extinguished when the light is turned off. The combination of SLS with psychedelics may also allow for a reduction in the dosage of psychedelic substances used in this context. It is important to note concerns about the safety and efficacy of psychedelic-assisted therapy (Petranker et al. 2020; Elk and Fried 2023; Kupferschmidt 2024), which may both undermine the motivation for SLS therapy if acute ASC experiences turn out to be not so impactful or increase this motivation by ameliorating safety concerns specific to psychedelics.

A small group of psychotherapists have been using SLS for the above purposes since the first wave of research into SLS. Psychotherapist Stanislav Grof tested SLS alongside psychedelics while developing protocols for psychedelic-assisted therapy and as an alternative following the prohibition of psychedelic substances (Grof 1996). Other practitioners and therapists have continued to explore the psychotherapeutic potential of compassionate use of SLS for various psychopathologies (Carr, personal communication 2024; Proeckl, personal communication 2024; Winkler, personal communication, 2024). These therapeutic approaches partly overlap with both historical and contemporary concepts in psychedelics-assisted psychotherapy, particularly the idea that psychedelic induced ASCs can serve as transformative experiences (Majić et al. 2015; Carr 2020). Finally, reports of SLS use within trauma therapy, specifically eye movement desensitization and reprocessing exist (Carr 2020). An additional application currently being explored is the use of SLS as a preparatory tool for psychedelic-assisted therapy. This approach offers the possibility to familiarize the patient with the therapeutic procedures and experience of ASCs, where SLS allows a fast termination of the experience in case of feelings of discomfort or adverse reactions. In this context, SLS serves to reduce concerns about the ability to cope with a challenging psychedelic experience and to train the patient to ‘open up’ and ‘let go’ when ASC experiences start to emerge. SLS has been started to be used in a clinical setting as a preparatory step before Ketamine-assisted therapy, although no systematic research on this subject has been published yet (OVID Clinics, personal communication, 2024). Rigorous research is needed to test the potential effects of SLS in a controlled manner, accounting for factors such as demand characteristics, expectancies, and the placebo effect.

### Phenomenology of experiences induced by SLS

Although the perceptual content of SLS-induced experiences was initially studied through introspection and qualitative measures, recent research has aimed to quantify SLS-induced VHs and ASCs. This shift seeks to develop a more precise understanding of the phenomenology of these experiences, and how they may be influenced by factors such as strobe frequency or inter-individual differences in personality. Contemporary qualitative methods that have been applied to SIVHs include interviews, textual responses, and drawings (Smythies 1959b; Beauté et al. 2025), while quantitative measures primarily rely on questionnaires developed in the context of psychedelic research, which assess ASC-related experiences (Studerus et al. 2010; Schwartzman et al. 2019a; Bartossek et al. 2021; Amaya et al. 2023) or tasks developed for the context of a given experiment (Allefeld et al. 2011; Shenyan et al. 2024). Some experiments have also incorporated psychophysics-like tasks to obtain more fine-grained perceptual data on specific perceptual qualities of SIVHs (Becker and Elliott 2006; Pearson et al. 2016).

**Figure 2:**
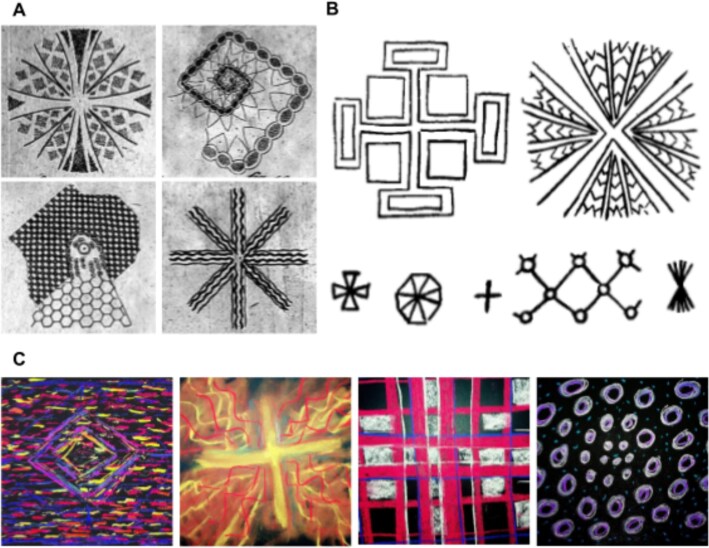
Visual depictions of geometric SIVHs. (A) Purkinje’s early drawings of SIVHs induced by waving his hands in front of his closed eyes while observing a candle flame (Purkinje, 1819). This appears to be the first published drawing of SIVHs, and more than a century would pass before any new drawings of SIVHs would be published. (B) Participant drawings of SIVHs, adapted from Smythies (1959b). (C) Selected drawings of SIVHs from participants in Dreamachine, courtesy of Collective Act.

The most universal aspect of the phenomenology of SLS-induced experiences is that of simple VHs, mostly consisting of geometric formations, a phenomenon readily replicable under laboratory conditions (Walter 1953; Smythies 1959b; Remole 1971; Becker and Elliott 2006; Becker et al. 2009; Shevelev et al. 2000; Allefeld et al. 2011; Sumich et al. 2018; Bartossek et al. 2021; Pari 2021; Rogers et al. 2021; Amaya et al. 2023; Montgomery et al. 2024; Shenyan et al. 2024). Figure 2 contains depictions of such VHs from the early 1800s to the present day. Complex VHs and other phenomena associated with ASCs including alterations to bodily perception, time perception, and emotions are also reported, though this is more common in cultural SLS contexts (Geiger 2004; Beauté et al. 2025) and is not always replicated under laboratory conditions (Bartossek et al. 2021).

### Phenomenology of simple geometric VHs

The majority of research investigating SLS visual phenomenology has focused on the prominent geometric aspects of these experiences. The most widely used classification system and theoretical frameworks for the geometric formations within simple VHs is Klüver’s form constants (see Fig. 1B; Klüver 1926) and the rotationally symmetric Turing patterns generated by neural field model simulations of simple VHs (see Fig. 1J and ‘Neural mechanisms’ section) (Ermentrout and Cowan 1979; Rule et al. 2011). However, research investigating the phenomenology of stroboscopic experiences consistently shows that participants report a broader variety of geometric forms than these frameworks can accommodate (Smythies 1959a; Shevelev et al. 2000; Bressloff et al. 2002; Beauté et al. 2025). For example, neural field models posit that all simple VHs should be organized around the centre of the visual field, where the highest level of detail occurs (Bressloff et al. 2002) (see ‘Neural mechanisms’ section). In contrast, phenomenological studies reveal a mix of rotationally and translationally symmetric forms such as grids and lattices (Smythies 1959b; Shevelev et al. 2000). Furthermore, SIVHs also appear to exist on a spectrum between regularly organized geometric formations (e.g. checkerboard) and non-geometric phenomena, such as visual noise, with many SIVHs being reported as falling somewhere between these two extremes (Smythies 1959b; Amaya et al. 2023). The current models and classification systems for simple VHs therefore fail to account for the range of SIVHs reported by hallucinators in phenomenological research, with a wider variety of geometric forms, visual noise, colour, and dynamics likely composing their phenomenological content.

SIVHs appear to change in content based on the frequency of stimulation. For example, Smythies found that doubling the SLS frequency mid-stimulation subjectively doubled the spatial frequency of the hallucinatory geometric pattern (Smythies 1959b). However, this relationship has not been systematically explored, partly due to the lack of an effective measure for the spatial frequency of hallucinatory content. The phenomenology of SIVH geometry may be linked to the alpha frequency band (8–12 Hz) within the brain. Numerous studies have found that geometric visual experiences peak around the alpha frequency range (Becker and Elliott 2006; Pearson et al. 2016; Bartossek et al. 2021; Amaya et al. 2023). When the stroboscopic stimulation frequency was matched to participants’ individual IAF, Shevelev and colleagues found that participants were more likely to report radial patterns at IAF compared to 1 Hz above or below IAF (Shevelev et al. 2000).

Another prominent aspect of the visual phenomenology of SIVHs is colour, with many individuals reporting colour as one of the most salient features of the experience (Smythies 1959b; Becker and Elliott 2006; Herrmann and Elliott 2001; Becker et al. 2009; Beauté et al. 2025). However, interpreting data on the specific colours experienced during SIVHs are challenging. The most common method of inducing SIVHs involves participants viewing the stroboscopic light through closed eyelids. Neutral or warm hues can be perceived when light passes through the eyelids due to the properties of blood and skin. Research investigating stroboscopically induced colour experiences using this method of stimulation has shown that lower stroboscopic stimulation frequencies (below alpha) tend to result in reports of neutral or warm colours, while frequencies at or above the alpha frequency range (8–12 Hz) are more likely to induce cooler colours such as blue, green, and purple (Amaya et al. 2023). The reported blues, greens, and purples are unlikely to be entoptic, and therefore more likely to be cortical in origin, while the warm and neutral colours experienced at lower frequencies may correspond to entoptic colours seen with constant light behind closed eyes, resulting from light passing through the eyelids.

Surprisingly little has been reported about the temporal dynamics of SIVHs—how hallucinatory content evolves and moves over time—despite these dynamic features being a common and salient aspect of reported experiences (Smythies 1959b; Beauté et al. 2025). Motion oriented around the centre of the visual field appears to dominate stroboscopic experiences, with participants frequently reporting rotational or spiral motion, as well as zooming in or out. Chaotic motion with no apparent pattern is also reported, along with more intricate transformations, such as one geometric pattern morphing into another (Smythies 1959b). Similar to reports of geometric formations, the perception of movement in SIVHs has also been reported to peak around the alpha frequency range (Amaya et al. 2023). In experiments using a strobing annulus, Pearson and colleagues found that increasing the strobe frequency resulted in faster perceived hallucinatory movement (Pearson et al. 2016).

### Phenomenology of complex VHs and ASCs

Outside of simple VHs, which dominate reports SLS-induced phenomenology in laboratory experiments, a variety of more heterogeneous experiences are reported, primarily in cultural contexts, which include other ASC phenomena such as complex VHs, out-of-body experiences, and emotional changes (Geiger 2004; Bartossek et al. 2021; Beauté et al. 2025). It remains debated if SLS indeed directly induces ASC phenomena outside of simple VHs, or if instead these phenomenologies have a distinct mechanism, potentially top-down in nature, or otherwise expectation-based, which may not be directly linked to the SLS itself.

While simple VHs consist primarily of colours, textures, and geometric formations which are most likely correlated with lower order areas of the visual system, complex VHs contain more semantic content with likely neural correlates in higher order cortical areas (Suzuki et al. 2017; Corlett et al. 2019). In the context of SLS, complex VHs are generally reported as being shorter in duration and combined with the more common simple VHs, such as a semantic object briefly appearing among the abstract hallucinated geometries. In cultural contexts, however, complex VHs that are more immersive, dramatic, and narrative in content have indeed been reported (Gysin and Wilson 1985; Geiger 2004; Sheehan 2008; Beauté et al. 2025). Complex VHs also appear less affected by frequency than simple SIVHs, including reports of complex VHs coming from experimental conditions consisting of SLS flashing outside the frequency range known to induce simple geometric VHs, such as 3 Hz (Schwartzman et al. 2019a; Bartossek et al. 2021). This lends some credence to the idea of complex SLS phenomena having a distinct mechanism. It is also difficult to disentangle complex VHs from pareidolia (Hodgetts 2017); naive hallucinators may describe or interpret simple VHs in semantic terms, such as by describing visual noise as ‘clouds’ or checkerboard patterns as ‘brick walls’.

Beyond hallucinations, a variety of other ASC phenomena have been reported during SLS, particularly outside of the laboratory. However, the occurrence of ASC phenomena during SLS is by no means a universal experience. Commonly, studies find that there is little or no ASC phenomena other than SIVHs experienced, or that only a portion of the tested population experience ASC phenomena (Bartossek et al. 2021; Schwartzman et al. 2019a). Such reported ASC phenomena include out-of-body experiences, emotional changes, euphoria, or changes to temporal perception (Geiger 2004; Bartossek et al. 2021; Beauté et al. 2025). As music or sound is often present during SLS, experiences of audiovisual synaesthesia or interactions between the visual and auditory experiences are sometimes reported (Montgomery et al. 2024; Beauté et al. 2025). SLS experiences have been described as somewhat resembling psychedelics-induced experiences without drugs, and as a potentially transformative therapeutic experience (Geiger 2004; Heller et al. 2023; Beauté et al. 2025). The nature of SLS-induced ASCs have been described as dream-like states or hypnagogia (Geiger 2004; Bartossek et al. 2021) and hypnotic states (Kroger and Schneider 1959; Hammer and Arkins 1964; Bause 2010).

Several factors may explain the discrepancy between the near universal reports of simple geometric hallucinations and less frequent reports of other ASC phenomena in laboratory settings. In cultural contexts, SLS often involves variations in frequency, duty cycle, ambient light, and light colours, which combine to create a more dynamic experience. Furthermore, these events typically pair SLS with music, which may enhance the emotional impact of the experience (Montgomery et al. 2024). Together these elements may contribute to the increased reports of ASCs at such cultural events, though no study has yet tested if such elements may induce ASCs where static strobe frequencies do not.

Other factors may contribute to experiences and reports of ASC phenomena. Firstly, demand characteristics giving rise to implicit or explicit participant expectations may lead participants to report having ASCs in contexts where they (implicitly or explicitly) believe they are supposed to experience them. Relatedly, specific psychological traits may contribute to the occurrence of ASCs in SLS, such as phenomenological control (PC), which refers to an individual’s ability to alter what they experience, both within and outside of the hypnotic context, in ways that are consistent with their plans and goals (Lush et al. 2023). In cultural stroboscopic events, participants’ expectations, shaped by advertising and the setting, may influence the nature of their experiences, including complex VHs and ASC. Few studies have tested the contribution of PC, psychological, or environmental factors on reports of stroboscopically induced ASCs. Recent studies indicate that participants who have higher absorption scores tend to report more intense ASCs (Bartossek et al. 2021). In the context of hypnosis, another phenomenon linked to PC, participants exposed to SLS typically did not report complex experiences unless primed to do so (Hammer and Arkins 1964); however, the role of expectancy has not been specifically tested in relation to ASC phenomena.

### Neural mechanisms of SIVHs

Over the decades, several theories have been proposed regarding the neural mechanisms of SIVHs. In the 1950s, Walter (1953) proposed that SLS induces VHs by disrupting how the brain supposedly scans across the visual field to construct visual experiences, a concept known as the ‘travelling wave hypothesis’; Costa (1953) theorized that SIVHs might be entoptic phenomena originating in the retina. However, more recent research has highlighted the role of primary visual cortex (V1) and thalamocortical interactions in the generation of SIVHs, with neural entrainment and higher-order brain areas also potentially contributing to these experiences. To date, the physiological mechanisms and neural correlates of SIVHs remain debated, with four theoretical frameworks reviewed here as the most prominent explanations, followed by a discussion how such theories may overlap or be synthesized to form a more nuanced view of the neurobiological mechanisms underlying SIVHs.

### Neural field model simulations

In the late 1970s, Ermentrout and Cowan formalized the idea that simple geometric VHs originate in lower order areas of the visual cortex through a theoretical model that demonstrated how patterns of activation in V1 could arise which resemble certain Klüver form constants commonly reported during VHs (Ermentrout and Cowan 1979). This model was later developed to incorporate additional anatomical features of the visual cortex, such as the diameter of cortical hypercolumns and their orientation selectivity (Bressloff et al. 2002; Cowan 2015). In 2011, Rule, Stoffregen, and Ermentrout applied these ideas specifically to SIVHs, demonstrating how a neural field simulation, when stimulated with a simulation of strobe light, could also produce geometric patterns which resemble some of Klüver’s form constants (Rule et al. 2011).

**Figure 3:**
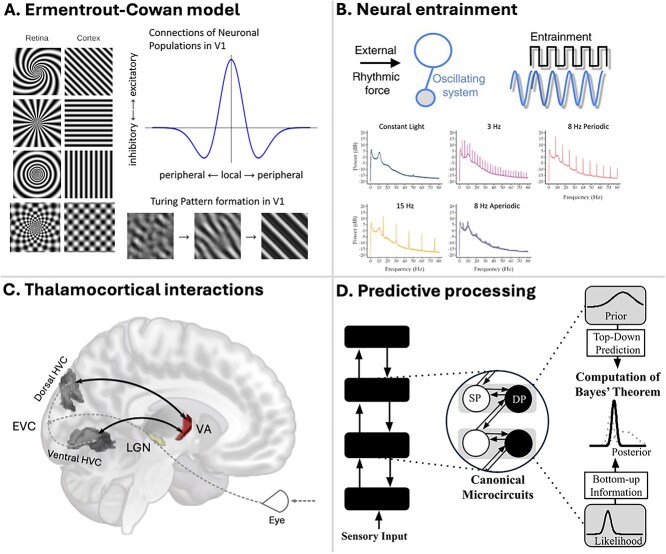
Four models of neural mechanisms and correlates of SIVHs. (A) Schematic of the Ermentrout and Cowan model of simple visual hallucinations (Ermentrout and Cowan 1979), later applied to SIVHs by Rule, Stoffregen, and Ermentrout (Rule et al., 2011). This model suggests that the visual cortex is predisposed to form spatially periodic patterns of neural activity (visualized in the four images labelled ‘cortex’ in panel A), which may give rise to percepts resembling Klüver form visual hallucinations of tunnels, targets, and spirals (see Fig. 1B). Images by Michael Rule and Ethan Grove. (B) Neural entrainment refers to the phase synchronization between periodic SLS and neural oscillations, which is most evident over visual areas of the brain. Top of Figure: Reprinted from Current Biology, Vol 29 Issue 28, Lakatos, Gross & Thut, A New Unifying Account of the Roles of Neuronal Entrainment, Page R891, Copyright 2019, with permission from Elsevier. (C) Recent fMRI studies show activity in higher order visual regions, as well as interactions between these regions and higher order thalamic nuclei (specifically the ventral anterior thalamic nucleus; VA), as the main correlates of SIVHs. (D) Predictive processing models suggest that phenomenal experiences result from comparisons between internally generated predictions (top-down) and sensory inputs (bottom-up). SIVHs may result from distorted approximate computation of Bayes’ theorem, due to rhythmic bottom-up input to the canonical microcircuits, potentially causing timing sensitive imbalances in cortical excitation and inhibition. In this context, individual differences in SIVHs may be explained by the variations in the influence of top-down signals, specifically in the weight or precision of priors.

These models of simple VHs propose that dynamical interactions between local excitatory neural connections and long-range inhibitory connections lead to repeating Turing patterns across V1 (Fig. 3A). While these dynamics generate laterally symmetrical patterns of V1 cortical activity, these models suggest that these patterns are perceived as the radial tunnel, funnel, and spiral Klüver form constants due to the way that visual information undergoes a spatial transformation upon entering V1. Neural field models of simple VHs therefore assume that the translationally symmetric patterns of activity in V1 (see images labelled ‘cortex’ in Fig. 3A) are perceived as being concentrated and organized around the centre of the visual field (see images labelled ‘retina’ in Fig. 3A). However, this observation relies on the potentially oversimplified assumption that activity in V1 corresponds to phenomenal experiences, at least somewhat directly, which may not be the case. Furthermore, this model does not account for other commonly reported patterns with different geometric properties, such as the Klüver form constant of checkerboards, lattices, or honeycombs or more complex geometric formations reported such as kaleidoscopes or fractal-like forms (see ‘Phenomenology of simple geometric VHs’ subsection) (Smythies 1959b; Shevelev et al. 2000; Bressloff et al. 2002). While in their original formulation of this model, Ermentrout and Cowan suggest that such laterally symmetric forms may only exist in the periphery of simple VHs, this claim is not supported by phenomenological data of the content of simple VHs (see ‘Phenomenology of simple VHs’ subsection).

While the models are able to produce geometric imagery which bears suggestive similarity to the kinds of geometric formations reported during SLS, the limited neural and phenomenological evidence that exists on this topic often diverges from their more specific predictions. Gulbinaite aimed to measure the patterns of visual cortex activity during SLS using a mouse model and found that standing wave patterns were generated exclusively within visual cortex (Gulbinaite et al. 2024). While the finding that geometric patterns form within visual cortices in response to SLS supports the notion that neural field models may explain certain aspects of SIVH content, the neural field models predict periodically repeating Turing patterns, not the standing wave patterns which best modelled Gulbinaite’s results. Furthermore, the neural field models predict that the spatial frequency of hallucinatory geometric forms will decrease as the stroboscopic frequency increases (Rule et al. 2011), the patterns measured in the mouse visual cortex exhibited the opposite relationship. Phenomenological data about VHs in humans may be more aligned with the results of Gulbinaite’s experiment, such as Smythie’s observation that an increase in the spatial frequency of a hallucinatory pattern could be induced by suddenly increasing the frequency of the SLS (Smythies 1959b). Additionally, Rule Stoffregen, and Ermentrout's neural field model further predicts two stable intervals of stroboscopic frequencies that would induce SIVHs, yet generally, only one is observed (Becker and Elliott 2006). While these models have provided initial insights into how SIVHs may arise from cortical activity, other factors are likely involved in the formation of these experiences, such as the influence of neural processes in other areas of the brain and retina, as well as circuit-level neural interactions.

### Neural entrainment

Neural entrainment in the context of SLS refers to the phase synchronization between a periodic stream of external flashes and neural oscillations, as measured using EEG. The most pronounced effect of SLS detected using EEG, over visual areas of the brain, is the steady-state visual evoked potential signal (Thut et al. 2011; Notbohm et al. 2016), which has been demonstrated to result from entrainment (Notbohm et al. 2016). The two main effects of neural entrainment due to SLS are an alteration in the phase of neural oscillations to match the phase of the SLS, and an increase in the amplitude of the power spectra at the stimulation frequency (Thut et al. 2011; Haegens and Zion Golumbic 2018). Despite neural entrainment being a prominent and well-researched neural effect of stroboscopic light, a mechanistic explanation which links neural entrainment to SIVHs remains elusive (Herrmann 2001; Thut et al. 2011; Notbohm et al. 2016; Amaya et al. 2024).

One approach to investigating the relationship between neural entrainment and SIVHs involves experiments comparing the subjective effects of periodic and frequency-matched aperiodic SLS. These experiments have demonstrated that frequency-matched aperiodic stimulation substantially reduces reports of SIVHs compared to periodic stimulation (ffytche 2008; Amaya et al. 2024). However, these studies have so far not assessed the neural impact of aperiodic stimulation, making it impossible to definitively establish the relationship between neural entrainment and SIVHs. While these results provide preliminary evidence suggesting that neural entrainment may contribute to the generation of SIVHs, the direct relationship between neural entrainment and the experience of SIVHs remains to be established.

### Thalamocortical interactions

fMRI studies suggest that hyperconnectivity between thalamic nuclei and cortical regions associated with higher order visual information processing plays a key role in the generation of SIVHs (Amaya et al. 2023, 2024). Specifically, experiments using frequency-matched periodic and aperiodic SLS in the alpha frequency range revealed that periodic stimulation increases both reports of SIVHs and thalamocortical hyperconnectivity between ventral thalamic nuclei and higher-order visual cortices, compared to aperiodic stimulation. The strength of the hyperconnectivity was found to correlate with the self-reported intensity of the stroboscopic subjective experience. SLS may specifically target the ventral matrix of the thalamus, which has widespread projections to supragranular cortical layers and is thought to contribute to the regulation of conscious experience (Phillips et al. 2019; Suzuki and Larkum 2020; Whyte et al. 2024b). Indeed, several theories of consciousness, such as dendritic information theory and the dynamic core theory, propose that thalamocortical interactions are crucial for generating the states and contents of conscious experience (Purpura and Schiff 1997; Tononi and Edelman 1998; Alkire et al. 2008; Ward 2011; Aru et al. 2019). This is likely due to the role of the thalamus in regulating cortical excitability (Honjoh et al. 2018; Shine 2021) and integrating information across cortical functional networks (Hwang et al. 2017), enabling it to act as a central hub that orchestrates activity across the brain (Bell and Shine 2016; Hwang et al. 2017; Whyte et al. 2024b).

In addition, the high spatial resolution of fMRI allows for the differentiation of specific areas along the cortical visual hierarchy that are activated by SLS (ffytche 2008; Amaya et al. 2024). fMRI studies have shown that periodic SLS results in stronger activation within the lateral occipital regions, which are shape and orientation selective (Grill-Spector et al. 1998; Malach et al. 1995; Silson et al. 2013) and V3b, which is motion selective (Zeki et al. 1991). Given that SIVHs are primarily composed of moving shapes and patterns, it is likely that this stronger activation in shape- and motion-selective areas correlates with the experience of SIVHs.

To date, the relationship between these fMRI findings and neural entrainment remains unclear. While fMRI studies indicate clear increases in connectivity between lateral geniculate nucleus (LGN) and V1, these alterations in connectivity did not differ significantly between periodic and aperiodic SLS (Amaya et al. 2024). In addition, changes in activation were not observed in early visual cortices, but rather in higher-order regions. Given that the low spatial resolution of EEG does not provide detailed information about the precise neural regions in which entrainment occurs, improved source localization of the oscillatory activity associated with entrainment could offer valuable insights in this context.

Furthermore, it is possible that the generation of SIVHs is not solely driven by input from the LGN to the visual cortex but also by the timing of thalamocortical feedback loops. For example, LGN projections to V1 may exhibit a frequency-dependent depression of excitatory postsynaptic potentials in response to SLS, depending on overall brain state (anaesthetized or awake) (Reinhold et al. 2015). Since higher-order thalamocortical projections have been shown to mediate the transition between anaesthesia and wakefulness (Redinbaugh et al. 2020; Suzuki and Larkum 2020; Bastos et al. 2021), visual cortical neurons may be influenced by functionally distinct thalamocortical pathways. In particular, the dynamics of higher-order projections may modulate the frequency dependence of geniculocortical response patterns (Reinhold et al. 2015). Therefore, the interaction between different thalamocortical feedback loops may collectively contribute to the phenomenal experiences associated with SIVHs.

### Predictive processing models

Over the past decades, predictive (or Bayesian) models of the brain have gained significant attention as a theoretical framework for explaining cortical information processing, including several formulations aimed at understanding the computational or dynamical principles underlying hallucinations (e.g. Fletcher and Frith 2009; Sterzer et al. 2018; Corlett et al. 2019). Central to these models is the idea that the brain’s primary function involves continuously generating predictions about the causes of sensory events (Friston and Kiebel 2009), with prediction errors—discrepancies between expected *versus* actual sensory input—driving learning and perception. The brain learns regularities about the world and uses this knowledge to internally generate predictions of the most probable upcoming sensory inputs, which are transmitted as top-down signals to lower-order cortical regions. The integration of these top-down priors with bottom-up sensory signals enables the brain to approximate Bayesian inference about the causes of afferent sensory input (Friston and Kiebel 2009; Seth 2022; Seth and Bayne 2022; Whyte et al. 2024a). Most interpretations of this process posit that perceptual content is given by the approximate posterior or ‘best guess’ (Seth 2022; Seth and Bayne 2022; Whyte et al. 2024a), which in normal circumstances (typical perception) can be informally described as a ‘controlled hallucination’. During typical perception, this process optimally combines prior knowledge with potentially uncertain and ambiguous sensory data to form useful, coherent percepts. However, various inductions of ASCs have been proposed to disrupt the balance between top-down and bottom-up signalling, leading to altered sensory processing, distorted perception, and the manifestation of hallucinations (Fort et al. 2024; Rieser et al. 2024). Among several examples, Corlett et al. (2019), Sterzer et al. (2018), and Suzuki et al (2024) developed computational models that propose how altered integration of top-down and bottom-up information could result in hallucinations.

Although no specific formulation of the mechanisms of SLS within the predictive processing framework has been proposed, it is plausible that SLS could disrupt the computation of prediction errors. Adams, Bastos, and Friston formulated canonical predictive coding microcircuits that have been proposed to approximate Bayes theorem within the functional architecture of a cortical column (Bastos et al. 2012; Adams et al. 2015). Within this framework, the rhythmicity of the driving bottom-up SLS input may disrupt the natural balance of excitation and inhibition within a given neuronal population, potentially shifting the system to allow an excessive influence of prediction errors, which could result in hallucinatory percepts.

Another possible mechanism is that the disturbance in visual information processing arises from inputs and interactions with non-sensory regions. During SLS, predictive processing could be disrupted by excessive thalamo-cortical input from higher-order thalamic nuclei to the cortical visual system. The strength and rhythmicity of these signals could interfere with the computation of prediction errors, potentially triggering the experience of SIVHs. Indeed, the matrix thalamus is thought to play a central role in top-down processing (Reinhold et al. 2015; García-Cabezas et al. 2019; Bastos et al. 2021). This speculative mechanistic hypothesis could be investigated through computational simulations of thalamo-cortical feedback loops in response to rhythmic input.

Moreover, predictive processing models provide a powerful framework for explaining between-subject differences in perceptual experiences in terms of variations in hierarchical processing. Predictive processing allows for individuals to differ in many aspects—e.g. in the precision of prior beliefs—which would directly influence how sensory data is interpreted. In this context, prior beliefs can be strongly shaped by expectations related to the experimental environment. Stronger expectations may enhance the precision of these priors, which in turn, could alter the perceptual outcome.

More generally, when predictive models fail to reconcile incoming sensory data with prior expectations, alterations in hierarchical computations may lead to phenomena such as ASCs or hallucinations (Sterzer et al. 2018; Corlett et al. 2019). In the context of SIVHs, the strength and nature of these hallucinations may be influenced not only by the precision of priors but also by an individual's level of PC (see ‘Phenomenology’ section). Individuals with higher PC may be more prone to consciously or unconsciously influencing the content and intensity of their hallucinations through more heavily weighted expectations. As such, expectancy and PC may be key factors in shaping the subjective effects of SLS, contributing to the diversity of experiences observed between individuals.

### Towards a unified theory of SLS

The various perspectives described here regarding the neural consequences of SLS may co-exist and interact with each other. For example, an electrophysiological study highlighted that phase-locking (entrainment) occurs in neurons of the LGN, the first relay for visual inputs, during SLS (Schneider et al. 2023). This suggests that neural entrainment may be initiated in the thalamus before exerting downstream cortical effects. Given that the periodicity of SLS is crucial for many aspects of stroboscopically induced phenomenal and neural effects (ffytche 2008; Amaya et al. 2024), it is possible that neural entrainment may serve as the driver for subsequent effects including SIVHs. Entrainment may trigger the perturbation of excitatory and inhibitory activity in early visual cortices, as described by the Ermentrout–Cowan model, leading to downstream activation of higher-order visual cortices *via* feedforward pathways, as revealed by fMRI, enabling the conscious representation of the dynamic geometric forms to crystallize. Alpha band (8–12 Hz) entrainment has been posited as a driver for SIVHs, though this remains a topic of debate. Several studies have reported that SIVHs tend to be more intense when SLS is delivered within the alpha band. However, SIVHs can also be reliably induced by stimulation outside the alpha band (Becker and Elliott 2006; Allefeld et al. 2011; Amaya et al. 2023). Qualitative differences in the hallucinatory experiences were reported when SLS is matched to intrinsic individual alpha peak, compared to stimulation 1–2 Hz above or below it (Shevelev et al. 2000). This is consistent with research highlighting the importance of the alpha band in the visual system (Başar 2012; Clayton et al. 2018; Helfrich 2018). Nonetheless, since SIVHs are reliably induced across a broader frequency range (~ 5–50 Hz), it is unlikely that alpha band oscillations alone are the only contributing factor to the generation of SIVHs.

In addition, hyperconnectivity between ventral anterior thalamic nuclei and higher-order visual cortices may indicate a modulation of neuronal excitability in the visual cortex *via* matrix thalamic innervation. This could lower the perceptual threshold, increasing the likelihood of hallucinatory percepts (Aru et al. 2019; Takahashi et al. 2020). Future research could test this hypothesis using effective connectivity analysis to identify the directionality of connectivity changes between regions. Since the matrix thalamus also regulates arousal (Whyte et al. 2024b), its recruitment may also underlie the additional phenomenal effects of SLS, such as sleepiness and an altered sense of time perception, as well as other ASC experiences (Bartossek et al. 2021). Finally, predictive processing may provide an encompassing framework within which these various neurophysiological processes can be understood as underpinning perceptual content during SLS, as well as accommodating individual differences and participant expectancies.

A deeper synthesis of the perspectives described here into a comprehensive model of stroboscopically induced neural effects and their role in generating SIVHs represents an important avenue for future research.

### Future outlook

Investigations into the subjective experiences induced by SLS face many open questions. To advance our understanding of this topic, future research should focus on improved characterization of the phenomenology of and neural mechanisms underlying SIVHs. Developing validated phenomenological measures that can capture the full range of SIVH is a crucial step in this process. Additionally, future work should aim to bridge the gap between empirical and theoretical phenomenological research by connecting more precise characterizations of SIVHs with models able to simulate the wide-range of visual features reported. This approach will help refine models of SIVHs, which in turn, can be used to elucidate their underlying neural origins, which can then be tested empirically. Clinical studies are also necessary to determine the efficacy and limitations of SLS for therapeutic applications.

Research can also benefit from the insights gained from the use of SLS in recreational settings. Reported experiences with SLS vary widely, from overwhelmingly intense to minimal and boring (Beauté et al. 2025). In recreational contexts, creatives working with SLS have access to a wide range of parameters—such as brightness, frequency, duty cycle, wash light, music, and environment—which can be adjusted to create experiences of varying intensities (Collective Act 2020; Light Attendance GmbH 2024). In laboratory experiments, experiential intensity has shown some variation, but stroboscopic frequency and luminance have been the only factors consistently linked to stronger subjective experiences. Further research should explore which combinations of stroboscopic parameters produce the most intense experiences and vivid VHs. Such studies could investigate the effects of adjusting the less-explored aspects of the stroboscopic light or the use of more dynamic strobe sessions from recreational SLS, alongside investigations into the contribution of psychological factors, such as expectancy and PC, which may play a significant role in shaping the subjective outcomes of SLS experiences.

There is currently a renaissance of interest in SLS research. There is considerable untapped potential to explore. Stroboscopic and neuroimaging technologies are advancing, and applications and exhibitions that provide hallucination-inducing SLS are becoming increasingly successful and creatively developed. Simultaneously, multiple laboratories internationally are working with SLS to investigate the nature of VHs and consciousness more broadly. SLS has already proven to be a valuable tool for exploring the phenomenology and neural mechanisms of VHs and potentially ASCs. At the same time, it remains a fascinating type of VH in its own right with deep roots in both culture and experimental scientific research.

## Glossary

**Altered States of Consciousness (ASCs)**: A transient state in which a person experiences significant changes from their usual conscious experiences, such as feelings of unity, altered bodily sensations, spiritual feelings, or anxiety (Fort et al. 2024). In this review, the term ‘ASC phenomena’, refers to alterations in different aspects of consciousness beyond the visual effects of SLS.

**Demand Characteristics:** Subtle cues or signals in an experimental setting that inform participants about the expected or desired behaviour, potentially influencing their responses and impacting the validity of the results.

**Entoptic Phenomena:** Visual effects that originate within the eye itself, such as floaters or visual snow, rather than from external visual stimuli.

**Expectancy:** A psychological state involving anticipation or belief about what will happen in a given situation, which can influence perception, behaviour, and subjective experience.

**Flicker Fusion:** The frequency at which stroboscopic light stimulation is perceived as a steady, constant light by the human eye.

**Neural Field Equations:** Mathematical models that describe the dynamics of neural activity across the cortex, helping to simulate and understand patterns of brain activity underlying perception.

**Phenomenological Control (PC):** The capacity of an individual to intentionally influence or alter their subjective experiences according to their expectations, intentions, or goals.

**Phenomenology:** The study of subjective experiences and consciousness from the first-person perspective.

**Stroboscopically Induced Visual Hallucinations (SIVHs)**: Non-veridical visual experiences, primarily consisting of colours and geometric patterns, which can be rapidly induced in healthy individuals through exposure to bright flashing light at periodic intervals between ~ 5 and 50 Hz.

**Stroboscopic Light Stimulation (SLS)**: Exposure to bright flashing light which may produce SIVHs.

**Turing Patterns:** Spatially periodic patterns that emerge from reaction–diffusion systems, used in neural field models to explain the formation of geometric visual hallucinations in the brain.

**Visual Hallucinations (VHs):** Visual experiences that do not correspond to external stimuli, deviating from an individual’s baseline waking perception.
